# The Microscope in Medicine

**Published:** 1891-03-14

**Authors:** Frank J. Wethered


### The Microscope in Medicine.

By Frank J. Wethered, M.D.

IX.—EXAMINATION OF THE SPUTUM (*continued*).

In this article it is intended to describe briefly what experience has shown to be the most rapid and reliable methods of staining the more important micro-organisms met with in sputum.

The tubercle bacillus naturally occupies the first place place. Too much stress cannot be laid upon the importance of obtaining a proper sample. This should be the expectoration coughed up in the early morning before any food has been eaten. It should be thrown out on to a black dish and search made for small opaque portions, about the size of hemp-seeds, which are so common in phthisical sputa. Failing these, any other opaque portions should be removed and gently pressed between two cover-glasses, any excess that may escape being wiped away with a soft cloth. The glasses must then be separated by a sliding movement, and not "sprung" apart, otherwise the layers of sputum will not be even. Two films, one on each glass, are thus obtained, which are allowed to dry exposed to the air. The glasses, prepared-side uppermost, are then passed rapidly three times through the flame of a spirit-lamp in order to coagulate the albumen and fix the films to the glass. If the last process be performed too slowly the film will be observed to become yellow and present fissures running across it; the specimen under these circumstances is useless, and a fresh one must be prepared.

It is occasionally rather difficult to ascertain on which side of the glass the film is. This may be determined either by gently scratching the surface and watching whether any mark is made, or by holding it towards the light and observing whether there is a reflection from the glass or not, the latter, of course, demonstrating the presence of the film. The glasses are now ready to be stained. In the opinion of the author the following is the most reliable and rapid method. Many practitioners use Gibbes's double stain; this is perhaps a little more simple, but not quicker, and not so much to be depended on for accurate results. The preparations are first floated on Neelsen's solution: Fuchsine, 1 part, dissolved in a 5 per cent. watery solution of carbolic acid, 100 parts; alcohol, 10 parts. A small quantity of this stain is placed in a watch glass and heated until steam begins to rise; the cover-glasses are then floated on it, prepared-side downwards, for *two minutes*. The best way to float the specimens is to hold each between the fingers and thumb, and then approaching it to the surface of the stain to drop it flat upon it.

After removal from the stain the glass is immersed for about *five seconds* in 25 per cent. watery solution of sulphuric acid, contained in a shallow capsule, and is next thoroughly washed in water. The colour, which disappears after swilling in the acid, is partially restored. If this assumes a more marked tint than a delicate pink the glass must be replaced for a few moments in the acid, and then again washed in water, this process being repeated until the film is almost colourless. The next process is that of counter-staining. This is accomplished by floating it on a solution of methyline blue having the following composition: Methyline blue 2 parts, alcohol 15 parts, water 85 parts. The immersion should be continued for *one minute*, and after thoroughly washing in water, staining is complete. After drying by means of blotting paper the specimen may be mounted in balsam, or, if required only for diagnostic purposes in water, glycerine, or cedar oil. It is best examined by an oil immersion. The rationale of this method is that the tubercle bacillus possesses a capsule permeable to aniline dyes but resisting for a short time the action of dilute acids; consequently when, after staining fuchsine the glass is placed in the, acid the general mass of sputum and all the micro-organisms, except the tubercle bacilli, are immediately decolourised, whilst the last named retain a brilliant red colour. In staining by Gibbs's method, the cover glasses prepared in the manner above described are immersed for five minutes in his special stain, which has been warmed until steam commenced to rise, and are then washed in methylated spirit until no more colour can be removed.

Stained by either of the above methods, tubercle bacilli appear as short rods, of rather variable size, but having an average length of half the diameter of a red blood corpuscle They occasionally present a beaded appearance, due to the presence of spores. A very distinguishing characteristic is their arrangement in small groups, two or three bacilli lying crosswise, or at angles to one another. As regards the diagnostic significance of tubercle bacilli in the sputum, it may be safely inferred that a tubercular process is going on somewhere in the respiratory tract, *where* the stethoscope or laryngoscope will soon decide. Their numbers can hardly be said to be indicative of the acuteness of the disease, so much depending upon the selection of the sample. A negative result is of little value unless repeated, and careful examination be made for some weeks. As an illustration of this the writer examined the sputum of a patient (supposed to be tubercular) twenty times without finding any bacilli, whilst on the twenty-first occasion a few were discovered. A short time afterwards the patient died from milliary tuberculosis of the lungs. Hence, whilst a positive result is of the greatest value, a negative one is no proof that tuberculosis does not exist.

Another organism whose existence is often sought for in the sputum is the microbe of pneumonia. Various forms have been described, and it must be admitted that the subject needs further elucidation. It is probable that there are several micro-organisms which may cause the disease, and consequently their diagnostic value is small. Those most frequently seen take the form of round or oblong bodies, surrounded by a gelatinous capsule of corresponding shape. The most common variety consists of small round bodies arranged in pairs. They are best stained by Friedländer's method. Films of the sputum are prepared on cover-glasses in the same way as in searching for tubercle bacilli. After drying and passing through the flame of a spirit-lamp, they are placed, prepared side downwards, in a 1 per cent. solution of acetic acid contained in the capsule. After three minutes they are removed and allowed to dry. They are next stained in a solution of methyl-aniline-violet, prepared in the following manner: A few drops of aniline oil are put into a test-tube, which is then almost filled with water. The tube is then thoroughly shaken, and the resulting emulsion filtered through a double fold of filter-paper. Some of the filtrate is then placed in a watch-glass, and an alcoholic solution of methyl-violet added drop by drop until the mixture just becomes opaque. In this stain the glasses are allowed to remain for half-a-minute, and are then rinsed in water, allowed to dry, and mounted for examination. By this method the capsule is clearly demonstrated.

In staining sputum by any of the methods described above, besides the pathogenic organisms sought for, numerous microbes will be seen which have no bearing on the disease. These organisms take the form of rods or cocci, the latter being the most numerous. In phthisical subjects, the sputum often contains a variety known as the Micrococcus Tetragonus. It occurs in groups of four cocci, surrounded by a hyaline capsule, and collected together in colonies. This arrangement is characteristic, and will easily be recognised, but, as with the other septic organisms, it is of no diagnostic value.

In the rare disease of actinomyces the parasite characteristic of the disease has occasionally been found in the sputum. On careful examination small yellowish granules can be seen, which, when crushed under a cover glass, exhibit the clubs which form the recognisable part of the fungus. For the purpose of corroboration, other granules should be stained by the method of Gram, when the mycelial threads come into prominence. The method of staining by Gram, briefly described, is as follows: A solution of methylaniline-violet is prepared as previously described. In this the cover glasses remain for five minutes, and are then placed for one minute in Gram's solution. This has the following composition: Iodine 1 part, iodide of potassium 2 parts, distilled water 300 parts. The specimens are then washed in equal parts of methylated spirits and water until no more colour comes out. If they are by that time not quite colourless they are replaced for a few seconds in Gram's solution, and then again washed in the spirit. They are counter-stained in a dilute solution of eosin.

